# Supplementary material for: Extended topological valley-locked surface acoustic waves
Source: Nat Commun. 2022 Mar 14;13:1324. doi: 10.1038/s41467-022-29019-8 (PMC8921310; doi:10.1038/s41467-022-29019-8)
Supplement: Supplementary file 1 — Supplementary Information [file 41467_2022_29019_MOESM1_ESM.pdf]

Supplementary Information for Extended topological valley-locked surface acoustic waves

Supplementary Note 1: Material parameters used in COMSOL Multiphysics.

To make our simulation better in conformity with practical situation, the anisotropy and piezoelectricity of $LiNbO_3$ are also taken into consideration, with mass density: 4700 kg m^{-3},
elastic stiffness matrix:

$$c^E = \begin{Bmatrix} 2.02897e^{11} & 5.29177e^{10} & 7.49098e^{10} & 8.99874e^{9} & 0 & 0 \\ 5.29177e^{10} & 2.02897e^{11} & 7.49098e^{10} & -8.99874e^{9} & 0 & 0 \\ 7.49098e^{10} & 7.49098e^{10} & 2.43075e^{11} & 0 & 0 & 0 \\ 8.99874e^{9} & -8.99874e^{9} & 0 & 2.43075e^{11} & 0 & 0 \\ 0 & 0 & 0 & 0 & 5.99018e^{10} & 8.98526e^{9} \\ 0 & 0 & 0 & 0 & 8.98526e^{9} & 7.48772e^{10} \end{Bmatrix}$$

piezoelectric coupling tensor (C m^{-2}): matrix:

$$\varepsilon^S = \begin{Bmatrix} 0 & 0 & 0 & 0 & 3.69594 & -2.53384 \\ -2.53764 & 2.53764 & 0 & 3.69548 & 0 & 0 \\ 0.193644 & 0.193644 & 1.30863 & 0 & 0 & 0 \end{Bmatrix}$$

and permittivity tensor:

$$e = \begin{Bmatrix} 43.6 & & \\ & 43.6 & \\ & & 29.16 \end{Bmatrix}$$

The elastic parameters of the Ni pillars are density ρ_{Ni}=8,906kgm^{-3}, Young's modulus E_{Ni} =175×10^9Pa and Poisson's ratio 0.31.

Supplementary Note 2: Sample preparation.

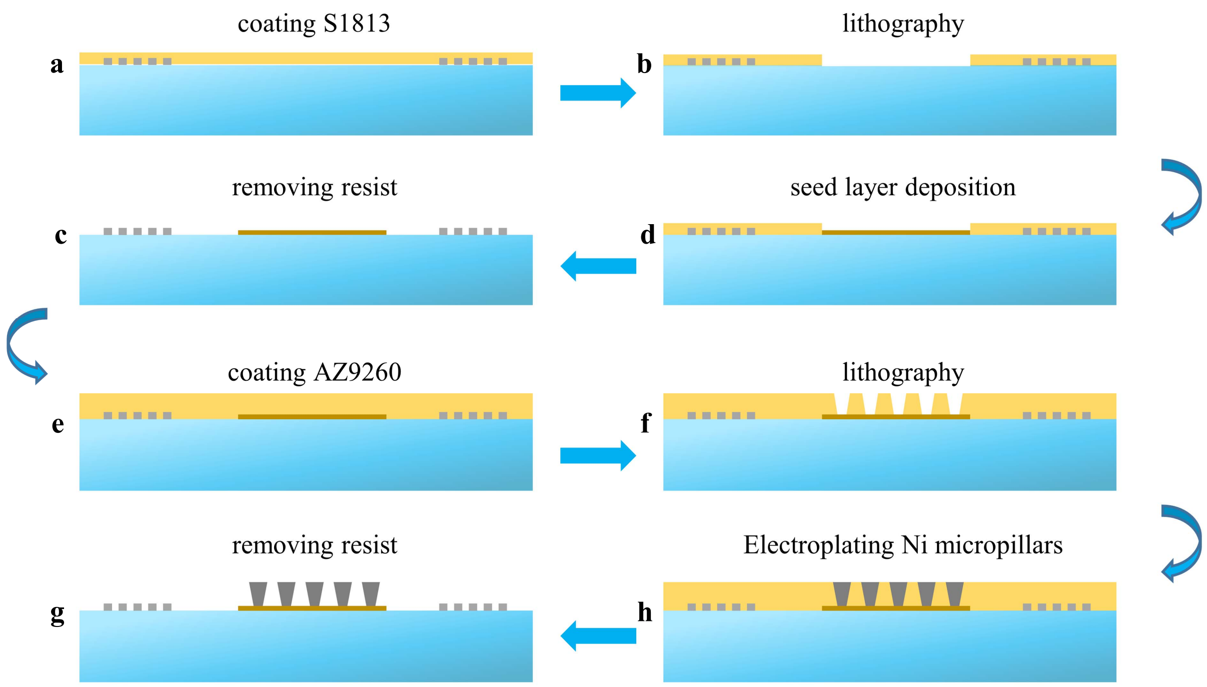

Figure S1 | Preparation process for our SAW phononic crystal. a: spin coating of photoresist (S1813, ~1μm). **b**: UV lithography. **c**: seed layer deposition. **e**: photoresist removing (S1813). **f**: spin coating of photoresist (AZ9260, ~10μm). **g**: UV lithography. **h**: electroplating for nickel (Ni) micropillars. **i**: photoresist removing (AZ9260).

SEM images of the fabricated SAW PnC samples
(all scale bars represent 50μm)

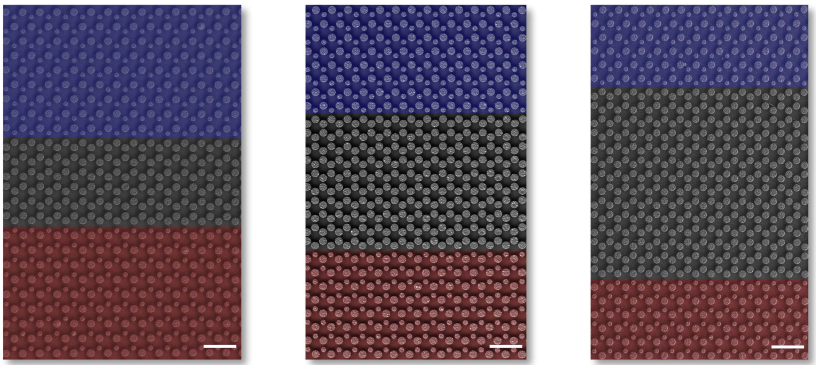

Figure S2 | Samples in Figs. 2d, 2e, and 2f

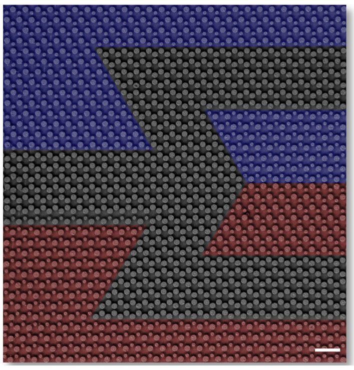

Figure S3 | Samples in Fig. 3

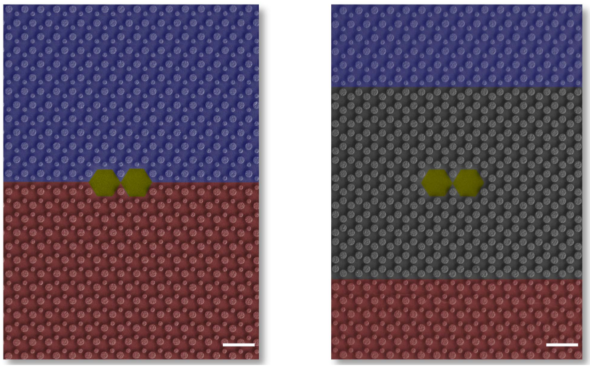

Figure S4 | Samples in Figs. 4a and 4b

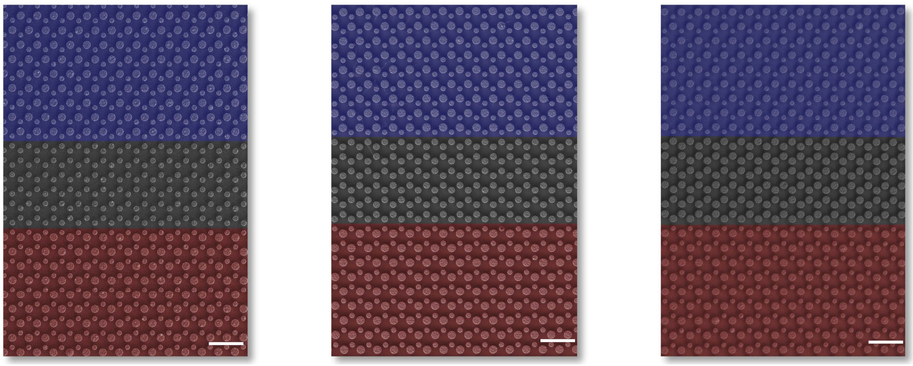

Figure S5 | Samples in Figs. 5a, 5b, and 5c

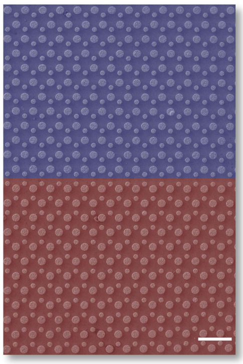

Figure S6 | Samples in Fig. 8c

Supplementary Note 3: Complete band structures for Fig.1

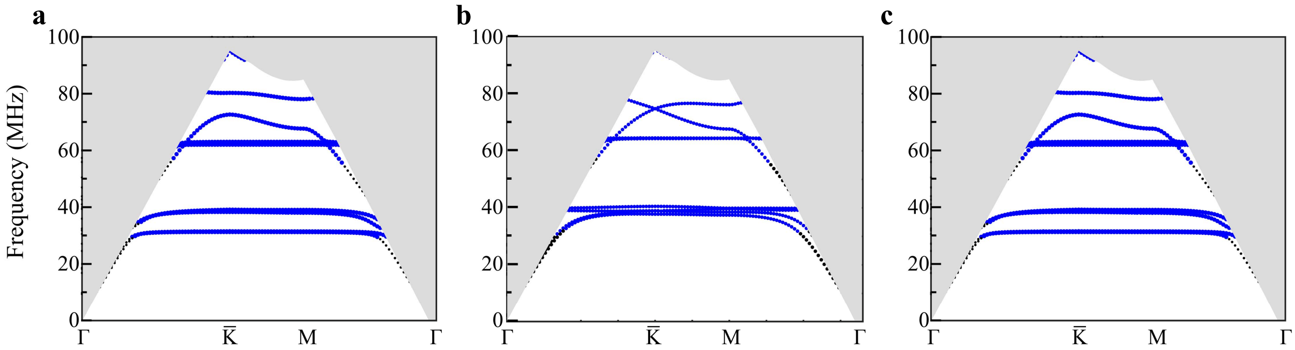

Figure S7 | Complete band structures for the SAW Phononic crystal. **a**: band structure of VA. **b**: band structure of SM. **c**: band structure of VB. The gray areas indicate the sound cone.

Supplementary Note 4: Berry curvature calculation of our phononic crystal.

The topological nontrivial characteristics of our SAW system is protected by the nozero valley Chern number(C_V), whichi is the integration of the Berry curvature around K and K' valley:

$$C_V^{(n)} = \frac{1}{2\pi}\int \mathbf{\Omega}(\mathbf{k})\, d^2\mathbf{k}$$

here $\mathbf{\Omega}(\mathbf{k})$ is the Berry curvature of the point with wavevector of $\mathbf{k}$, and:

$$\mathbf{\Omega}(\mathbf{k}) = i\nabla_k \times \langle u(k)|\nabla_k|u(k)\rangle$$

$\mathrm{u}(\mathbf{k})$ denotes the normalized displacement field of the eigenmode. For numerical discretizat, when calculation patches are much smaller than the first Brillouin zone, in our case, $\delta k_X = \delta k_Z = 3.8e^3\ m^{-1}$ (shown in Fig. S8) and the periodic length of the first Brillouin zone is $2.6e^5 m^{-1}$, $\mathbf{\Omega}(\mathbf{k})$ can be rewritten as[1,2]:

$$\mathbf{\Omega}(\mathbf{k}) = \mathrm{Im\ In}\,[\boldsymbol{U}^{(n)}_{\mathbf{k}_1\to\mathbf{k}_2}\boldsymbol{U}^{(n)}_{\mathbf{k}_2\to\mathbf{k}_3}\boldsymbol{U}^{(n)}_{\mathbf{k}_3\to\mathbf{k}_4}\boldsymbol{U}^{(n)}_{\mathbf{k}_4\to\mathbf{k}_1}]$$

where: $\boldsymbol{U}^{(n)}_{\mathbf{k}_\alpha\to\mathbf{k}_\beta} = \frac{\langle \mathbf{u}(\mathbf{k}_\alpha)|\mathbf{u}(\mathbf{k}_\beta)\rangle}{|\langle \mathbf{u}(\mathbf{k}_\alpha)|\mathbf{u}(\mathbf{k}_\beta)\rangle|}$, $\alpha, \beta = 1, 2, 3, 4$, and $\mathbf{k}_1, \mathbf{k}_2, \mathbf{k}_3, \mathbf{k}_4$ are the vertices (black poins in Fig. S8) of the each plaquette.

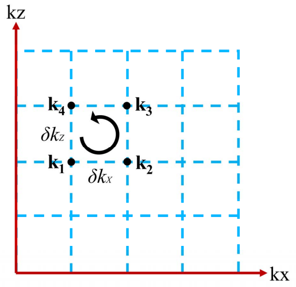

Figure S8 | computational scheme for the Berry curvature.

Supplementary Note 5: SAW valley flux vortices of our phononic crystal.

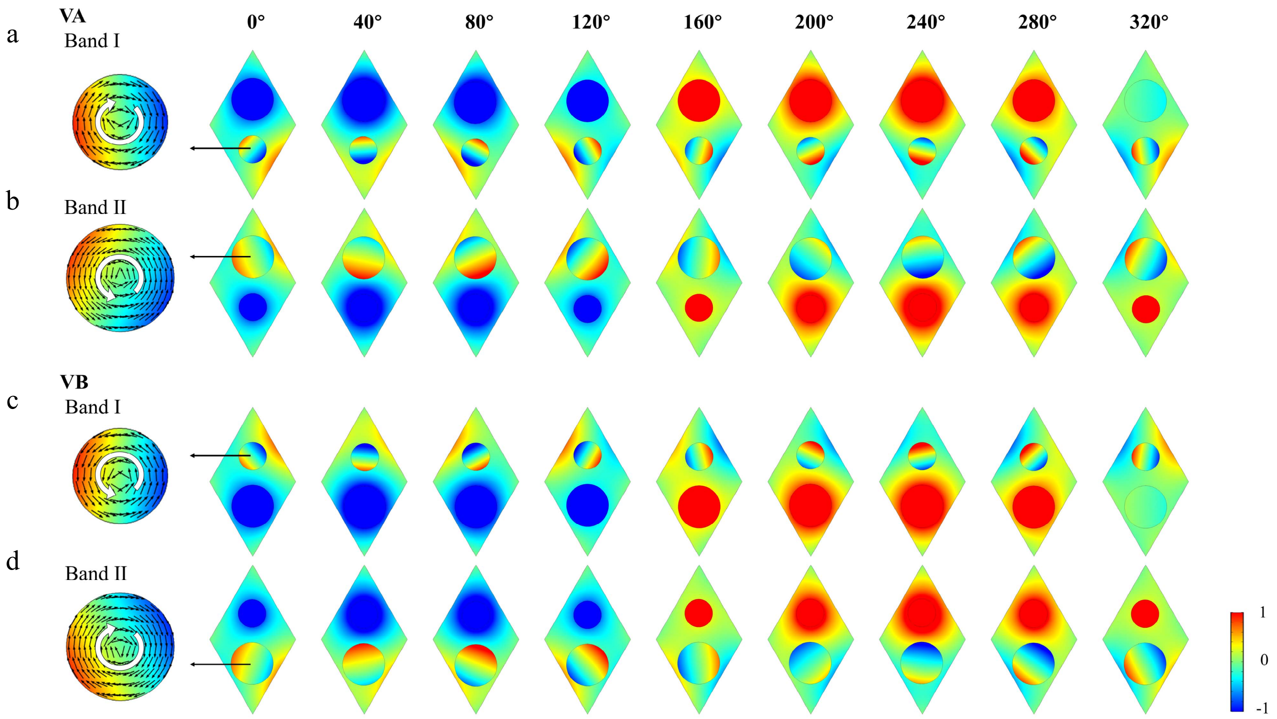

Figure S9 | SAW flux vortices of our phononic crystal. **a** and **b**: in our SAW insulator A (VA), time-dependent out-of-plane displacement fields for SAW modes at the valley ($\bar{K}/\bar{K}'$ point) from bands I and II. The SAWs around the micro-pillars exhibits vortex characteristics but have opposite chirality for the two bands. **c** and **d**: in our SAW insulator B (VB), time-dependent out-of-plane displacement fields for SAW modes at the same valley from bands I and II. Comparing VA and VB, the opposite chirality switched for the two bands.

Supplementary Note 6: Observation of SAW quantum valley Hall Effect

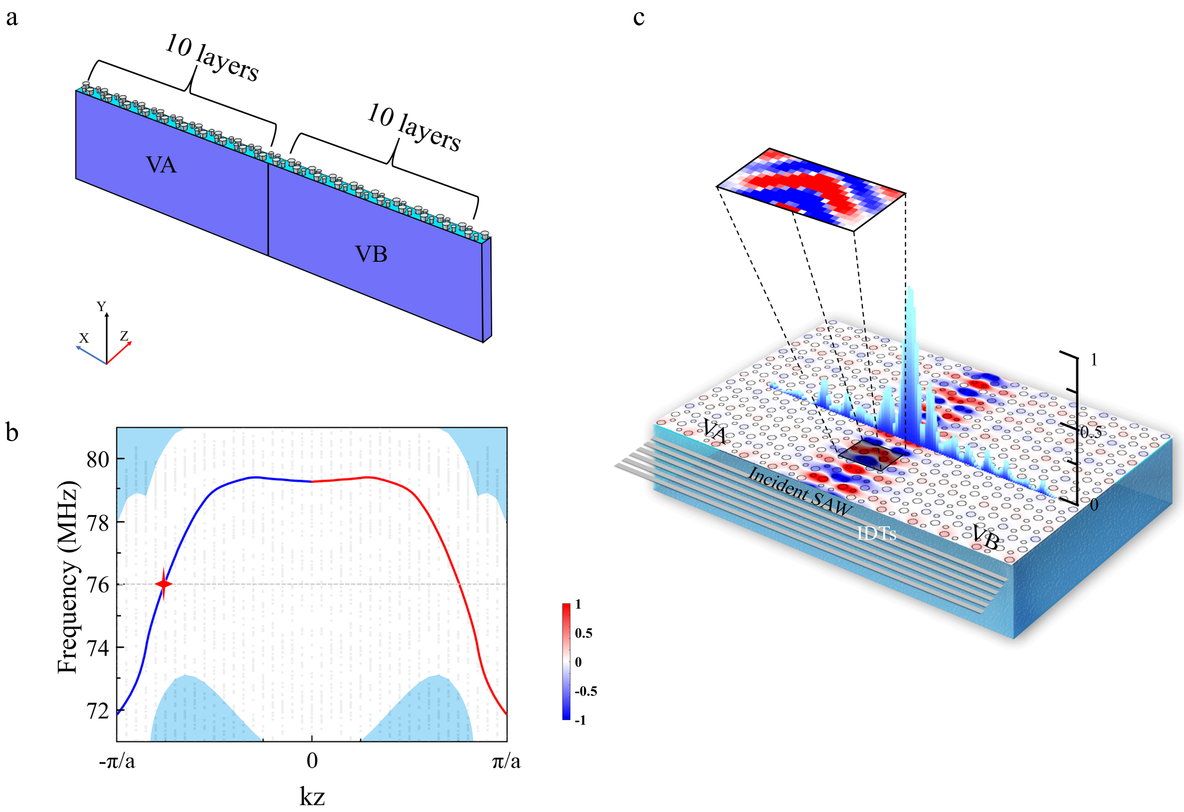

Figure S10 | Observation of analog quantum valley Hall Effect (QVHE) for SAWs on a $LiNbO_3$ substrate. a: supercell for the simulation of the SAW edge states, consisting of adjacent VA and VB. **b**: simulated band structure of the valley-locked edge states. **c**: (bottom) simulated and (upper) experimentally measured SAW energy and displacement fields around the VA-VB interface at the frequency of 76MHz.

Supplementary Note 7: $LiNbO_3$ substrate (isotropic v.s. anisotropic)

The trigonal crystal of $LiNbO_3$ slightly affects the symmetry of our honeycomb (in appearance) phononic crystal, making it not strictly C6v. However, this effect is faint and does not affect the topological properties of the phononic crystal.

First, to show the influence of the anisotropy of LNO on the 2D phononic crystal, we calculated its band structures and equal frequency contours (EFCs) without considering anisotropy (also without piezoelectricity). The results are shown in the upper panel of Fig. S11. The Young's modulus, density, and Poisson's ratio of this hypothetical material are set to 203GPa, 4700 kg m^{-3}, and 0.31, respectively (upper panel). For comparison, the lower panel of Fig. S11 shows the results of real $LiNbO_3$ (both anisotropy and piezoelectricity are taken into account).

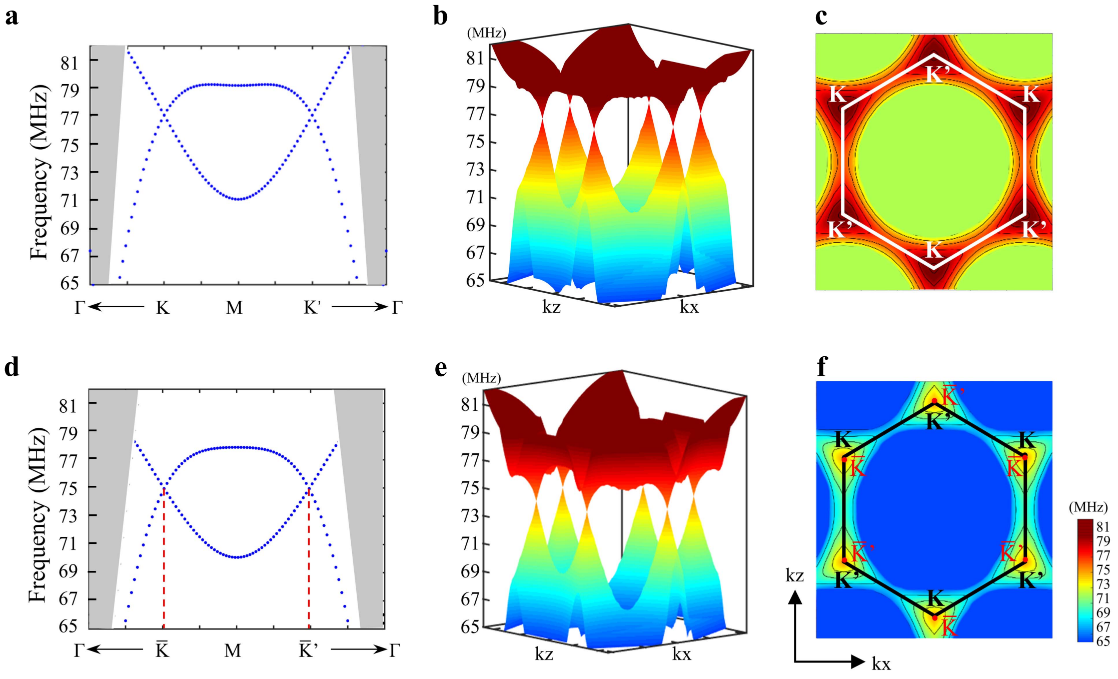

Figure S11 | Band structures and EFC with and without consideration of LNO substrate's anisotropy and piezoelectricity . Band structure without anisotropy and piezoelectricity **a**: band structure along Γ-K-K'-Γ. **b**: 3D band structure of the 1st BZ. **c**: EFC of the 1st BZ. Band structure with anisotropy and piezoelectricity **d**: band structure along Γ-$\overline{K}$-$\overline{K}$'-Γ. **e**: 3D band structure of the 1st BZ. **f**: EFC of the 1st BZ

In the isotropic $LiNbO_3$, the SAW Dirac points of the phononic crystal are strictly located at the high-symmetric K and K' points of the C6v lattice. In real $LiNbO_3$ with anisotropy and piezoelectricity, the SAW Dirac points still exist, and the band structure has not changed much. Still, affected by the anisotropy $LiNbO_3$ substrate, the SAW Dirac points will be slightly offset along the z-direction in the EFC diagram. We use $\overline{K}$and $\overline{K}$' to indicate the positions of SAW Dirac points at this time. The distance between $\overline{K}$ and K is only about 1/100 of the distance between Γ and K.

Second, to demonstrate that the anisotropy of $LiNbO_3$ will not affect the topological properties of the 2D phononic crystal, we further calculated the Berry curvature of the isotropic $LiNbO_3$ and the real $LiNbO_3$, as shown in Fig. S12. In both cases, the Berry curvature has a local distribution of equal magnitude and opposite

sign at the K($\overline{\text{K}}$) and K'($\overline{\text{K}}$') points, thus supporting the QVH states.

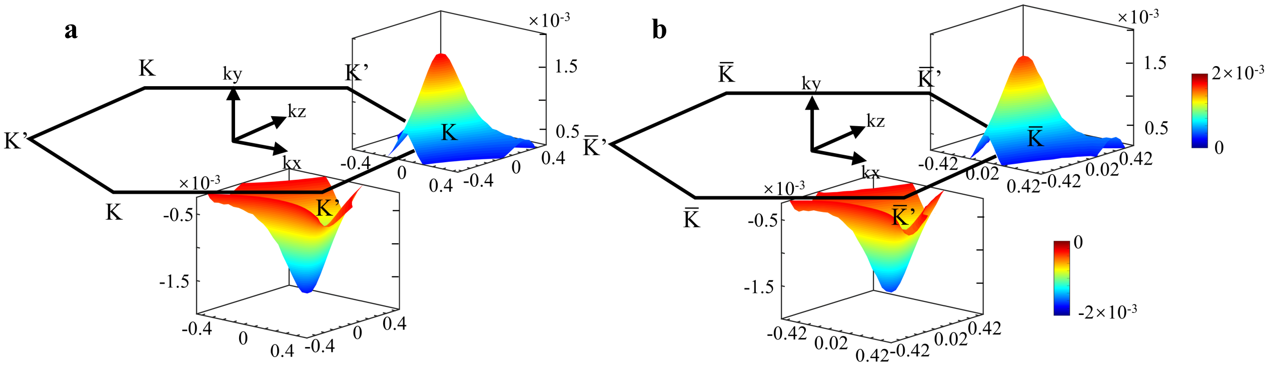

Figure S12 | Berry curvature with and without LNO substrate's anisotropy and piezoelectricity . **a**: Berry curvature around K and K' points under isotropy LNO. **b**: Berry curvature around $\overline{\mathbf{K}}$ and $\overline{\mathbf{K}}$' points under LNO substrate with anisotropy and piezoelectricity.

Third, we conduct some analysis on the formation of valley-locked edge states under the influence of LNO anisotropy, as shown in Fig. S13a shows the Zigzag interface formed by valley insulator A (VA) and valley insulator B (VB) in the real space. S13**b** and S13c show the Berry curvature at the VA-VB interface in the isotropic LNO case and the real LNO case, respectively. The distributions of Berry curvature at K ($\overline{\text{K}}$) or K' ($\overline{\text{K}}$') inversed when crossing the Zigzag interface, leading to nonzero valley-projected Chern number. Thus, valley-locked topological edge states will certainly appear along with the Zigzag interface under both circumstances, according to bulk-edge correspondence[3]. There are discussions about the effect of material symmetry on the symmetry of phononic crystals[4].

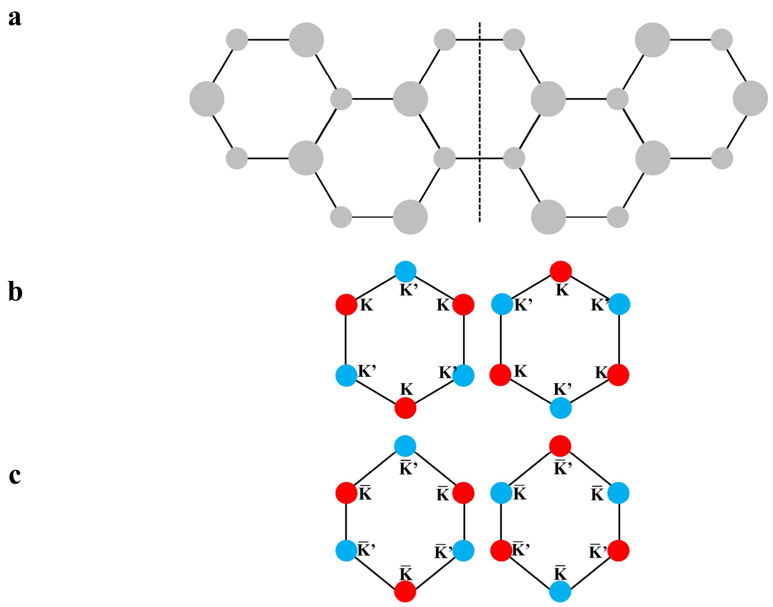

Figure S13 | Zigzag interface formed by valley insulator A (VA) and valley insulator B (VB) .
a: Zigzag interface formed by valley insulator A (VA) and valley insulator B (VB) in real space.

b: Berry curvature distribution in isotropy LNO on both side of Zigzag interface. **c**: Berry curvature distribution in anisotropy LNO on both side of Zigzag interface(to make the picture more clear, the offset distances from K(K’) to $\overline{\mathrm{K}}$ ($\overline{\mathrm{K}}$’) is enlarged 10 times).

Supplementary Note 8: Critical width of the ETVSs

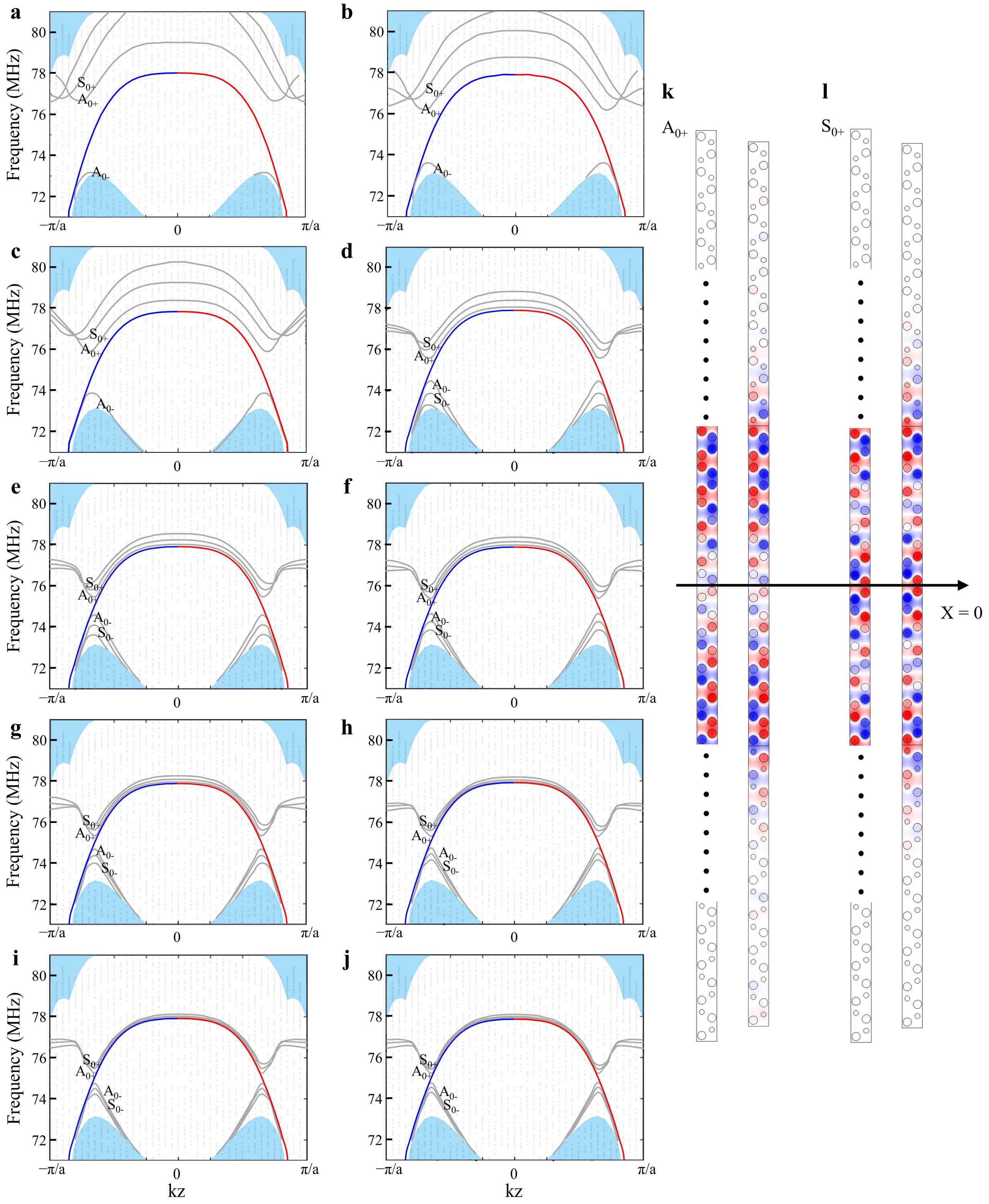

Figure S14 | Band structures of the SAW extended topological valley-locked state. a to **j**: band structures of the ETVSs from 3 to 39 doping layers, the step length is 4 layers. **k**: field distribution of A_{0+}. **l**: field distribution of S_{0+}.

In Fig.S14a to Fig.S14j, the ice-blue areas indicate the bulk bands, and the red/blue solid line is the dispersion of ETVSs. Solid gray lines represent the modes in the doping layer but are not protected by the valleys. They can be further divided into symmetric (*S*) and antisymmetric (A) modes, as shown in Fig.S14k and Fig.S14l. The high order S and A modes, e.g., $S_{1/2...}$ and $A_{1/2...}$, which do not influence the operating bandwidth, are hidden in the band structures for a clear presentation. With the increase of the doping layers, low order ordinary edge modes (e.g., $S_{0/1}$ and $A_{0/1}$ draw in the band structures) from the lower and higher bands will gradually get closer, and will finally turned into semimental bulk state.

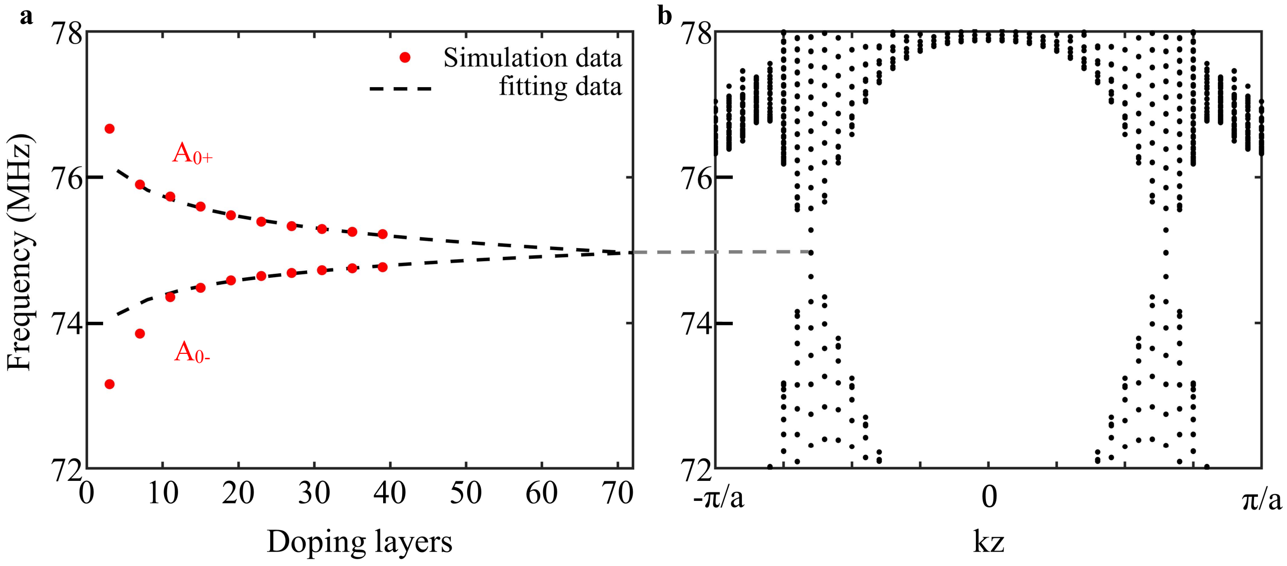

Figure S15 | Band structure evolution trend of the SAW extended topological valley-locked state. a: simulated gap of A_0 modes from 3 to 39 doping layers and corresponding fitting data. **b**: subject band structure of seminmental.

Based on the simulation data, we performed an approximate fit to the band structure evolution trend, as shown in Fig. S15. The fitting results show that when the number of doping layers reaches over 72, the A_0 bandgap may eventually close. The fitted closing frequency is about 74.94MHz, just the Dirac frequency of the semi-metal. It is reasonable because the Dirac semi-metal will gradually dominate the whole structure with the doping width increasing, where the interaction between two insulators becomes weaker and finally negligible. However, it should be noted that the exact value of the critical width of the semi-metallic region (*i.e.*, 72 layers in our simulation) highly depends on the boundary condition of the heterostructure (e.g., the width of the insulator besides the semi-metal). In the simulations of Figs. S14 and S15, both insulators besides the semi-metallic region are set as 8 layers. Moreover, with the change of the material and geometric parameters of the phononic crystal, this critical width may also vary to a certain extent.

Supplementary Note 9: maximum working bandwidth of the ETVSs

In our SAW system, the maximum effective working bandwidth of the ETVS is determined by two factors:

(I) the bulk bandgap opened by the A-B sublattice asymmetry

(II) the bandgap between S_{0-} and S_{0+}. The antisymmetric (A) modes are not taken into consideration because they cannot be excited by the IDTs.

The overlapping bandwidth of the I and II determines the working bandwidth of the ETVSs. To determine the maximum bandwidth of I, we calculated the distribution of Berry curvature at the $\overline{K}$ and $\overline{K}$' points of our phononic crystal when the A-B sublattice asymmetry gradually became stronger, as shown in Fig. S16

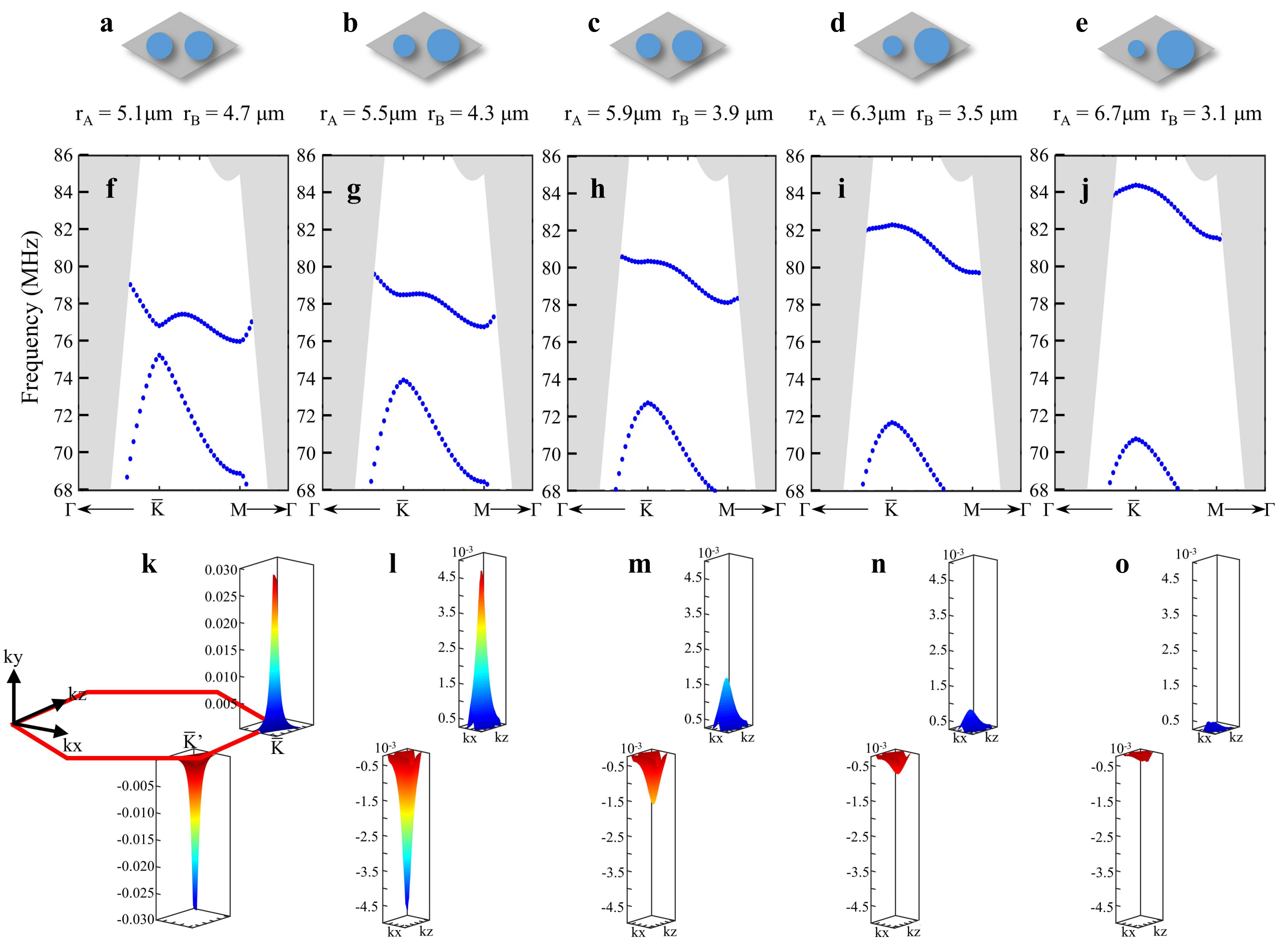

Figure S16 | Band structures and Berry curvature corresponding to different unit cell. a-e: phononic crystal unit cell with A-B sublattice asymmetry gradually becaming stronger **f-j**: phononic crystal band structure with A-B sublattice asymmetry gradually becaming stronger. **k-o**: Berry curvature distribution at $\overline{K}$ and $\overline{K}$'points with A-B sublattice asymmetry gradually becaming stronger.

Clearly, with the increase of A-B sublattice asymmetry, the intensity and localization of Berry curvature near the $\overline{K}$ and $\overline{K}$' points gradually decrease. In Fig. S16e/j/o, the maximum/minimum of Berry is only 1/60 of that in Fig. S16a/f/k Although the bandwidth at this time has exceeded 10MHz (~13%), the system's non-trivial

topological characteristics have become quite weak.

To find the maximum bandwidth of **II**, we calculated the projected band structures with different doping layers and increasing sublattice asymmetry, as shown in Fig. S17. Noticing S_{0-} mode in Fig. S17a/b/d/e/g is merged in bulk bands.

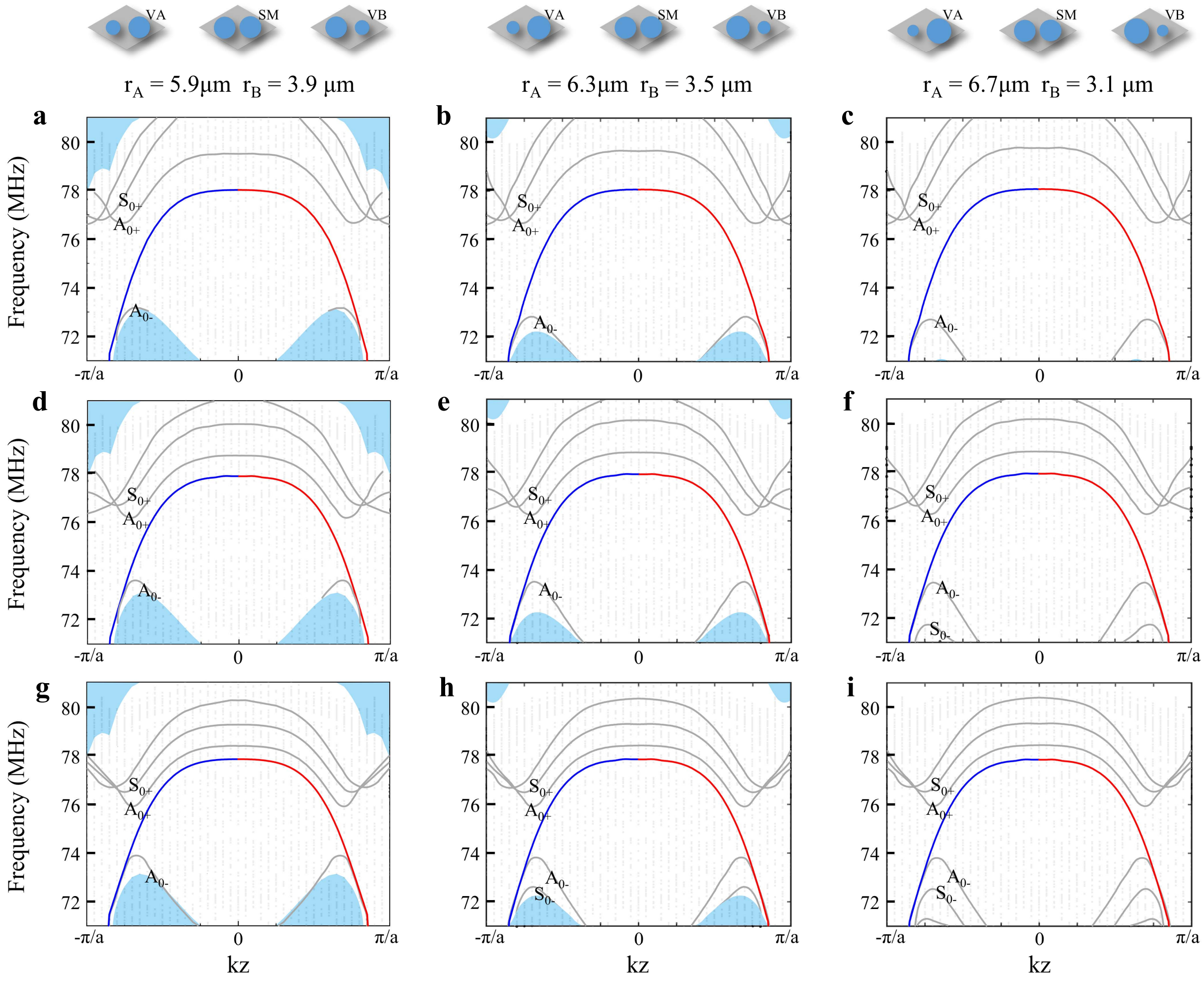

Figure S17 | subject band structure under different sublattice asymmetry. a, d, g: subject band structure of 3, 5, 7 layers doped ETVS with r_A = 5.9μm, r_B = 3.9μm. **b, e, h**: subject band structure of 3, 5, 7 layers doped ETVS with r_A = 6.3μm, r_B = 3.5μm. **c, f, i**: subject band structure of 3, 5, 7 layers doped ETVS with r_A = 6.7μm, r_B = 3.1μm.

When the number of doping layers is constant, the S0 bandgap is almost fixed; this value does not change with A-B sublattice asymmetry. This is understandable because the S0 mode exists in the doping area (i.e., the semi-metallic region), so it is hardly affected by the two insulators with the A-B sublattice asymmetry. Notably, when the A-B sublattice asymmetry is increased to a certain extent, the overlapping bandwidth of the I and II bandgaps is only determined by II. Therefore, in our SAW phononic crystals, for 3, 5, and 7-layer doping ETVSs, their maximum working bandwidths are 5.74MHz (7.8%), 5MHz (6.3%), and 3.97MHz (5.7%), respectively.

Supplementary Note 10:

Transmittance (S_{21}) comparation between TVs and ETVS

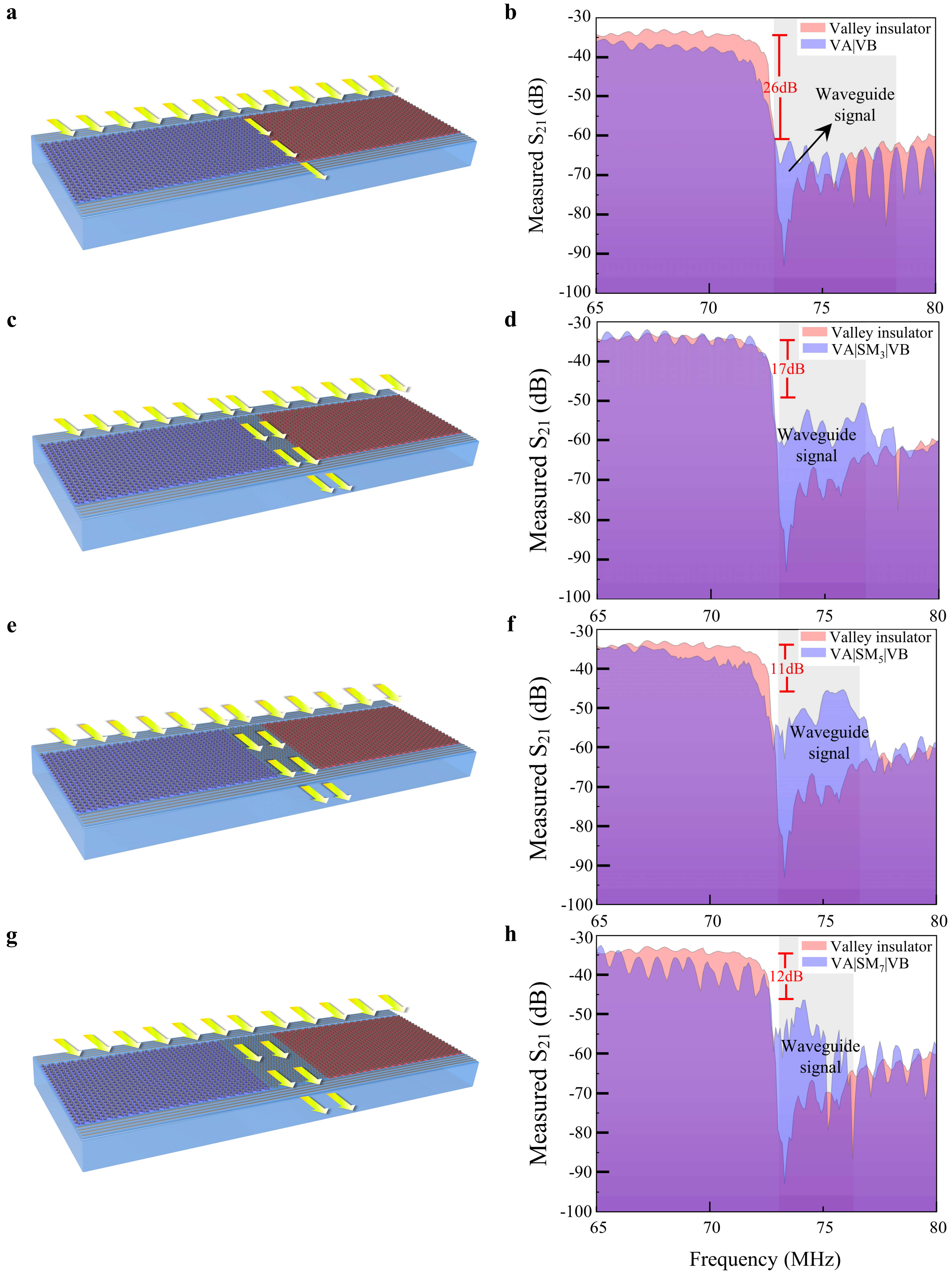

Figure S18 | measured transmittance (S_{21}) between the import and outport IDTs. a:

schematic diagram of topological valley-locked edge state(TVS), the yellow arrows indicat input and transmission surface acoustic waves, the red arrows indicate surface acoustic waves reflected by valley inslator(VA and VB). **b**: S_{21} of the TVS, the grey band indicates the effective operating band. In the operation band, SAW transmission of the waveguide is observed, but with relatively poor signal contrast and a huge (~26dB) S_{21} drop. Such a low S_{21} is not surprising because of the dramatic dimensional mismatch between the necessary wide aperture of the broadband IDTs and the narrow 1D SAW waveguide, *i.e.*, the VA-VB interface. This poor electro-acoustic-electric transmission performance severely limits the application potential of topological SAW waveguides. **c, e, g**: schematic diagram of VA-SM-VB heterostructured samples with doping of 3, 5, and 7 layers, respectively. **d, f, h**: S_{21} of the ETVS, comparing with the TVS one, we achieved considerable signal contrast, which is 10dB, 15dB, 14dB S_{21} improvements corresponding to 3, 5, and 7 layers doped ETVS respectively.

As we all know, SAW transducers with large bandwidth often require relatively large apertures. In the experiment, because we need to perform on-chip testing from 65MHz to 85MHz (about 30% bandwidth), the aperture of our transducer is very large (2.5mm, about 50-60 times the SAW wavelength). Consequently, only a part of the SAW electrically excited by the transducer can enter the waveguide for transmission, And finally be received by another transducer on the emitting end.——This whole process brought about the insertion loss (from 11dB to 25dB) that the reviewer mentioned.

Figs. S18a-b show the situation when the topological waveguide is not extended (widened). It can be seen that only a small part of the SAW excited by IDTs can enter the waveguide, so the insertion loss is large (~26dB), and the resolution of the signal transmitted by the waveguide is very poor (the blue area in the figure).

As a comparison, Figs. S18c-h show the situations after the topological waveguide has been extended (widened). At this time, a greater proportion of SAW can enter the waveguide, thereby considerably reducing the insertion loss. For waveguides with 3, 5, and 7 doping layers, the insertion loss is reduced by about 9dB (from 26dB to 17dB), 15dB (from 26dB to 11dB), and 14dB (from 26dB to 12dB) respectively. Although the current experimental data cannot show a quantitative relationship, it is enough to qualitatively demonstrate the advantages of these extended topological waveguides. They greatly match the topological waveguides and practical transducers for SAWs, thereby improving the resolution of the signal transmitted by the waveguides.

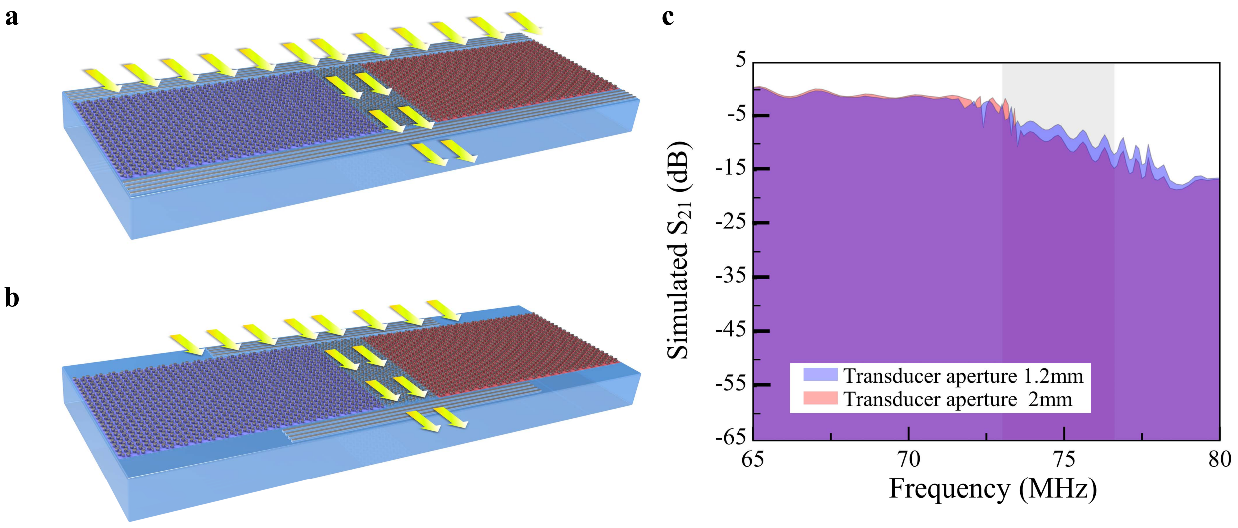

Figure S19 | Transmission comparation between different aperture IDTs. a, b: schematic diagram of 5 layers doped ETVS with a: ~2mm aperture IDTs b:~1.2mm aperture IDTs. **c**: transmission under ~2mm and ~1.2mm aperture IDTs.

When the aperture of the SAW waveguide is closer to the aperture of the transducer, this insertion loss will be further reduced. For example, we simulated the situation when the transducer aperture was reduced from 2mm (~50 times of wavelength) to 1.2mm (~30 times of wavelength), as shown in Fig. S19c For a waveguide with a certain width, when the transducer aperture is reduced to 60% of the previous one, in the S_{21} spectrum between the generating and receiving transducers, the signal brought by the waveguide is improved by about 3dB.

Supplementary Note 11: band structures of different doping radius in Fig.5.

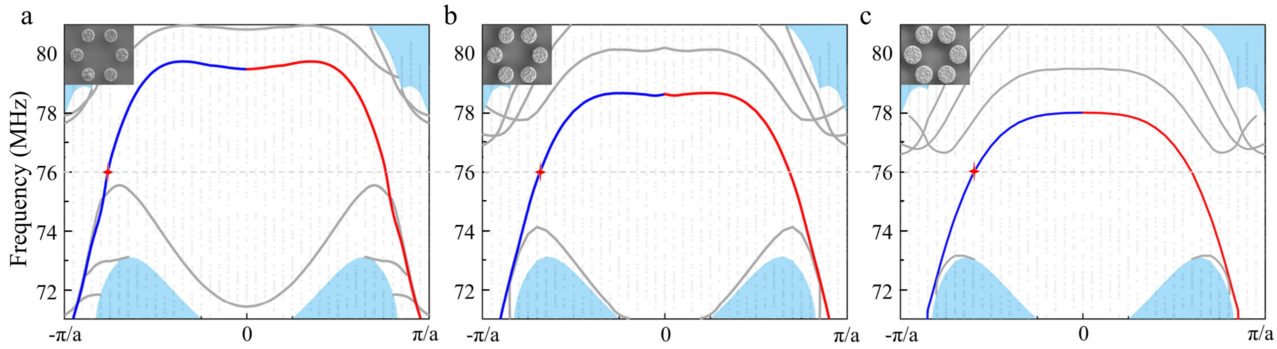

Figure S20 | Projected band structures correspond to different doping radius. **a** to **c**: projected bands with differently doped semimetal **a** ω_D=77.90MHz, **b** ω_D=75.99MHz, **c** ω_D=74.94MHz.

1 Zhao, R. *et al.* First-principle calculation of Chern number in gyrotropic photonic crystals. *Opt. Express* **28**, 4638-4649, (2020).

2 Bisharat, D., Davis, R., Zhou, Y., Bandaru, P. & Sievenpiper, D. Photonic Topological Insulators: A Beginner's Introduction [Electromagnetic Perspectives]. *IEEE Antennas and Propagation Magazine* **63**, 112-124, (2021).

3 Rudner, M. S., Lindner, N. H., Berg, E. & Levin, M. Anomalous Edge States and the Bulk-Edge Correspondence for Periodically Driven Two-Dimensional Systems. *Phys. Rev. X* **3**, 031005, (2013).

4 Li, S., Kim, I., Iwamoto, S., Zang, J. & Yang, J. Valley anisotropy in elastic metamaterials. *Phys. Rev. B* **100**, 195102, (2019).
